# Attributable risk and time trend in hemorrhagic and ischemic stroke mortality due to high sodium intake in Zhenjiang City from 2010 to 2021: an Age-Period-Cohort (APC) analysis

**DOI:** 10.3389/fstro.2026.1722772

**Published:** 2026-06-25

**Authors:** Xiaoyong Gu, Yuelan Zhu, Hongyu Wang, Lu Xu, Jiajia He

**Affiliations:** Zhenjiang Municipal Center for Disease Control and Prevention, Zhenjiang, Jiangsu, China

**Keywords:** APC analysis, attributable risk, high sodium intake, stroke mortality, time trend analyses

## Abstract

**Introduction:**

GBD studies have showed high sodium intake's attributable burden on stroke mortality, while existing risk data remain predominantly global, national, or provincial-level, lacking representativeness for specific cities or regional areas. By associating salt intake with elevated systolic blood pressure, it becomes feasible to conduct risk analysis attributing high sodium intake to specific population groups.

**Objective:**

To determine the attributable risk of stroke mortality due to high sodium intake in Zhenjiang City, Jiangsu Province, China, from 2010 to 2021, and analyze time trends in hemorrhagic and ischemic stroke mortality rates associated with high sodium intake, to provide a scientific basis for evaluating and improving the effectiveness of local dietary salt reduction policies.

**Methods:**

Using GBD data and Zhenjiang chronic disease surveillance records, this study calculated the attributable burden of hemorrhagic and ischemic stroke mortality caused by high sodium intake through a sodium intake-increase of SBP correlation method, referencing the death risk of elevated SBP leads to hemorrhagic and ischemic stroke. Joinpoint regression was employed to analyze mortality trend amplitude and direction, while an APC model evaluated age, period and cohort effects.

**Results:**

From 2010 to 2021, the PAFs for hemorrhagic and ischemic stroke death due to high sodium intake ranged between 12.0% and 19.3%, showing a yearly decreasing trend with higher amplitude observed in males than females. The AAPC of ASMR for hemorrhagic and ischemic stroke was −8.10% (95% CI: −12.00% to −3.90%), with hemorrhagic stroke was −11.10% (95% CI: −13.30% to −8.90%), and ischemic stroke ASMR demonstrated a downward trend after 2013, with its AAPC was −12.30% (95% CI: −14.80% to −9.70%). The overall net drift of mortality for hemorrhagic and ischemic stroke due to high sodium intake was below 0, with hemorrhagic stroke showing greater decline amplitude than ischemic stroke. The value of local drifts with age showed a trend that initially stabilizes and shifted to decreasing and then increasing. Although the age effects on female ischemic stroke mortality due to high sodium intake increased after age 85, both period and cohort effects show a downward trend in stroke mortality risks, the favorable and unfavorable cohort effects on different stroke mortality in different genders deserved more attention.

**Conclusion:**

In Zhenjiang City, the attributable risk of hemorrhagic and ischemic stroke mortality due to high sodium intake has shown a downward trend. Priority should be given to women and elderly populations, with continued implementation of salt-reduction-focused stroke prevention strategies to mitigate the health impacts of excessive sodium intake.

## Introduction

Stroke is classified into ischemic stroke caused by vascular occlusion and hemorrhagic stroke resulting from vessel rupture (Diener and Hankey, 2020). Although their pathogenesis differs, both ultimately lead to brain injury (Kalogeris et al., 2016; Chen et al., 2014), resulting in extremely high disability and mortality rates that have long imposed a heavy disease burden on society. Studies indicate (Wu et al., 2019) that China has over 2 million new stroke cases annually, with the condition accounting for the highest proportion of disability-adjusted life years (DALY) among all diseases. The latest Stroke Burden Study from GBD estimates (Feigin et al., 2023) that from 2020 to 2050, stroke-related DALY, mortality, and prevention costs will double. Evidence clearly links high sodium intake to cardiovascular and cerebrovascular diseases including stroke (He and MacGregor, 2010; Jayedi et al., 2019), which increases mortality through elevated SBP levels (He and MacGregor, 2002; Whelton, 2021; Filippini et al., 2021). Statistics show 92.6% of Chinese adults exceed recommended salt consumption guidelines (Fang et al., 2020). Research from the United States (Bibbins-Domingo et al., 2010) indicates reducing daily salt intake by 3 grams could prevent 32,000–66,000 new stroke cases annually.

GBD studies have showed high sodium intake's attributable burden on stroke mortality, while existing risk data remain predominantly global, national, or provincial-level, lacking representativeness for specific cities or regional areas. This study utilized GBD-related data and attributable disease burden calculation theory, combined with chronic disease surveillance data from Zhenjiang City, to calculate the attributable risk of hemorrhagic and ischemic stroke due to high sodium intake in the region from 2010 to 2021. We analyzed changes in mortality rates and their correlations with age, period, and birth group, and evaluated improvements in disease burden caused by stroke deaths due to high sodium intake and the effectiveness of salt-restriction-based stroke prevention measures comprehensively. We hope to provide scientific evidence for adjusting and implementing subsequent stroke-related health intervention policies.

## Methods

### Data sources

The primary data for stroke-related deaths in Zhenjiang City from 2010 to 2021 were obtained through three systems: the Disease Surveillance Point System (DSP), Maternal and Child Health Surveillance System (for verifying the causes of death and the number of deaths among pregnant women and children), and the Mortality Reporting System of the Chinese Center for Disease Control and Prevention (CDC) (Zhou et al., 2019). The medical classification of stroke follows the International Classification of Diseases (ICD-10), with hemorrhagic stroke coded as I60–I62 and ischemic stroke as I63. The calculation of attributable deaths from hemorrhagic and ischemic stroke due to high sodium intake in Zhenjiang City was based on the relative risk data from GBD 2015 (GBD 2015 Risk Factors Collaborators, 2016), which shows increased risks for each 10 mmHg rise in SBP across different age groups. This was combined with chronic disease and risk factor surveillance results, using a sodium intake-SBP (systolic blood pressure) correlation method. The world standard population was used to calculate ASMR (age-standardized mortality rate) for hemorrhagic and ischemic stroke due to high sodium intake.

Zhenjiang City conducted eight citywide surveillance programs for adult chronic diseases and risk factors in 2011, 2012, 2013, 2014, 2015, 2016, 2017, and 2020, demonstrating a highly sophisticated monitoring system. Using multi-stage cluster random sampling methods, the program collected 33,655 participants across these 8 years. The survey methodology integrated elements from U.S. behavioral risk factor monitoring and China's chronic disease nutrition surveillance, comprising three components: interviews, physical measurements, and laboratory tests. The findings were scientifically validated, comprehensive, and representative. Notably, daily salt intake was measured through 24-h dietary surveys using household condiment survey scales recorded over three consecutive days (two workdays and one weekend day). Blood pressure measurements followed the Chinese Hypertension Prevention and Treatment Guidelines (Wang, 2025), involving two measurements taken at more than 60-s intervals, recorded the average value. If the difference between two SBP readings exceeded 10 mmHg, a third measurement was required with recorded values.

## Statistical analysis

**Figure 1:**
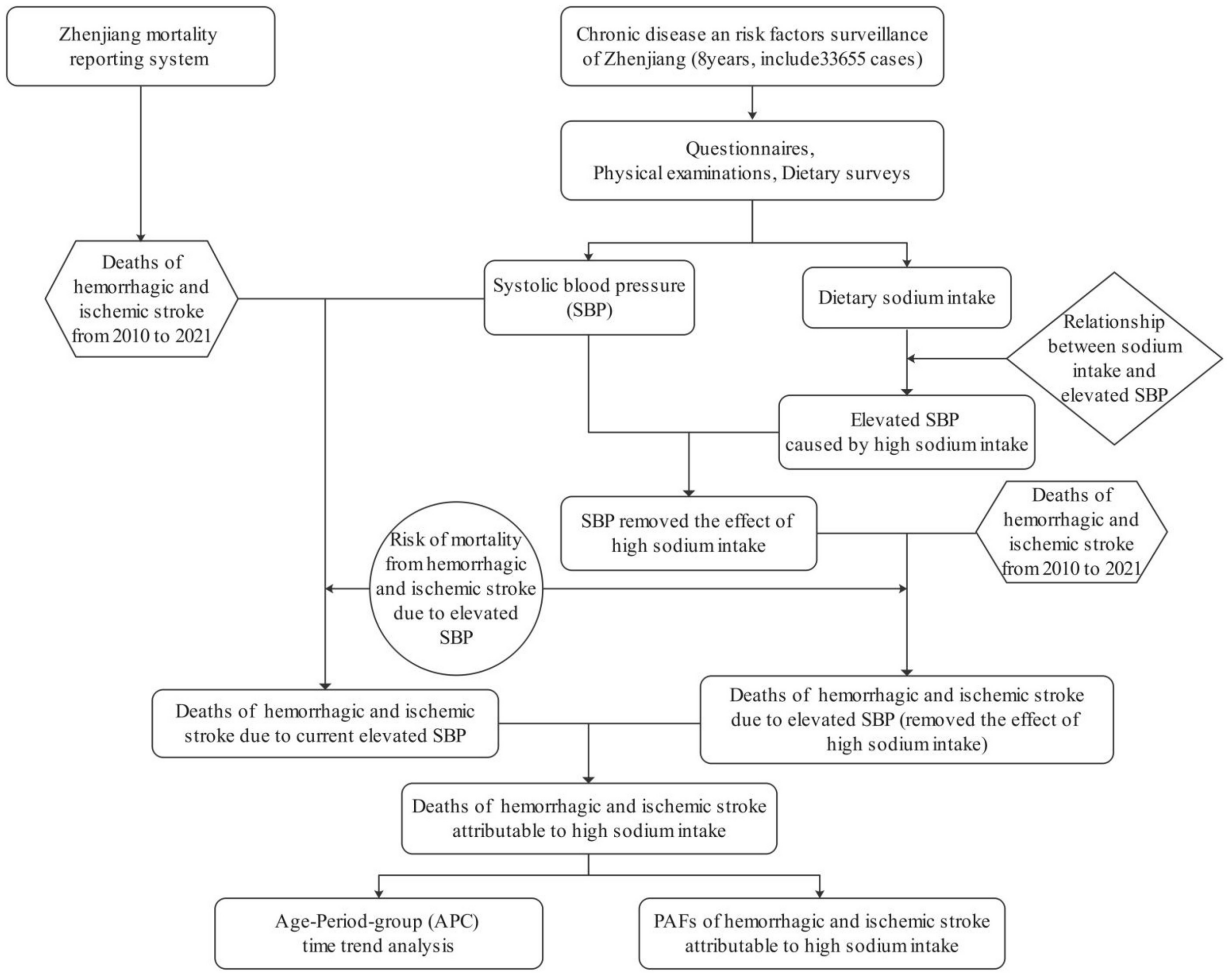
Flowchart of the study design.

The statistical analysis of this study was conducted in two steps, the flowchart of the study design is showed in Figure 1. Step 1: Following the methodology of Xue et al. (2023), we calculated annual mortality data for hemorrhagic and ischemic stroke attributed to high sodium intake in Zhenjiang City from 2010 to 2021, categorized by gender and age. The sodium intake-SBP correlation formulas (Mozaffarian et al., 2014) were used to calculated the SBP increase corresponding to each 2,300 mg/24 h sodium intake above the theoretical minimum (2,000 mg/24 h). We first calculate the increase in SBP levels resulting from elevated sodium intake across different age groups and years, then compare these with actual measured SBP data to determine SBP under sodium-free intake conditions and the population-wide exposure rate of SBP elevation (relative to 115 mmHg). By integrating data from the GBD on the increased risk of hemorrhagic and ischemic stroke mortality associated with elevated SBP in different age groups, we estimate the PAFs (population attributable fractions) for the risk of hemorrhagic and ischemic stroke mortality attributable to high sodium intake, subsequently calculating age-specific mortality data attributable to high sodium intake for different populations. **Step 2** involves applying the APC (Age-Period-Cohort) model to analyze the potential associations between the effect of considering age, period, birth cohort and the mortality from hemorrhagic and ischemic stroke due to high sodium intake. As cases of stroke-related deaths caused by high sodium intake in individuals under 45 years are rare (about 1.02% in our study), this study excluded data from this age group. The age effect refers to specific health risks in different age groups; the period effect denotes health impacts arising from particular events experienced during specific periods, such as relevant health policies or targeted interventions; the cohort effect indicates variations in health outcomes among individuals within the same birth cohort due to differences in lifestyle or occupational exposure. This study focuses on analyzing net drift, local drift, longitudinal age curves, period RR (risk ratio), and cohort RR within the model to explore the health effects of age, period, birth cohort, and their interactions on stroke mortality caused by high sodium intake. The general log-linear form (Wang et al., 2023) and calculation method (National Cancer Institute, 2014) for the APC model are detailed in Supplementary material 1.

The significance of parameters and functions were verified by Wald's chi-square test, and the *P* value of the double-tailed test was < 0.05 to be considered statistically significant.

## Results

### Basic information

Table 1 presents chronic disease surveillance data from Zhenjiang City covering the period 2010–2021, including dietary sodium intake, SBP levels, hemorrhagic stroke deaths, and ischemic stroke deaths. The daily sodium intake per capita ranged between 3349.4 g and 3812.7 g, with males at 3395.7 g−3784.0 g and females at 3309.6 g−3839.0 g. Average SBP level was range between 126.0 mmHg and 132.2 mmHg, with males at 128.0 mmHg−133.8 mmHg and females at 123.6 mmHg−130.8 mmHg. High sodium intake caused a 3.0 mmHg−3.5 mmHg increase in SBP level, with 3.1 mmHg−3.5 mmHg observed in males and 2.9 mmHg−3.5 mmHg in females. The number of deaths from hemorrhagic and ischemic stroke ranged between 400 and 607 cases, including 186–311 deaths from hemorrhagic stroke and 125–336 deaths from ischemic strokes. More detailed data by year, age and gender are listed in Supplementary Tables S1–S5.

**Table 1:** Summary of key surveillance data of hemorrhagic and ischemic stroke death caused by high sodium intake in Zhenjiang city from 2010 to 2021.

Year	Death			Sodium intake (mg/24h)			SBP increasing attributable to high sodium intake (mmHg)			SBP (mmHg)			Deaths attributable to high sodium intake		
	Hemorrhagic stroke	Ischemic stroke	Both	Mean	95%CI		Mean	95%CI		Mean	95%CI		Hemorrhagic stroke	Ischemic stroke	Both
					low	up		low	up		low	up			
2010	1,928	676	2,604	–	–	–	–	–	–	–	–	–	311	125	436
2011	1,762	952	2,714	–	–	–	–	–	–	128.5	128.0	129.1	287	169	456
2012	1,889	1,262	3,151	3,812.7	3,754.3	3,877.8	3.5	3.3	3.6	126.0	125.4	126.5	297	217	514
2013	2,021	1,571	3,592	–	–	–	–	–	–	127.8	127.4	128.3	305	258	563
2014	1,718	1,618	3,336	3,631.4	3,581.5	3,686.5	3.2	3.1	3.3	126.4	126.0	126.9	272	265	537
2015	1,806	1,886	3,692	3,446.6	3,393.1	3,502.5	3.0	2.9	3.1	127.7	127.4	128.2	282	325	607
2016	1,824	2,049	3,873	3,679.4	3,626.3	3,734.4	3.4	3.2	3.5	126.7	126.3	127.2	271	336	607
2017	1,646	2,165	3,811	3,744.2	3,696.1	3,793.0	3.3	3.2	3.4	127.4	126.9	127.8	233	329	562
2018	1,517	1,450	2,967	–	–	–	–	–	–	–	–	–	214	232	447
2019	1,472	1,466	2,938	–	–	–	–	–	–	–	–	–	204	238	442
2020	1,482	1,395	2,877	3,349.4	3,304.8	3,389.8	3.3	3.2	3.5	132.2	131.8	132.6	199	235	434
2021	1,427	1,438	2,865	–	–	–	–	–	–	–	–	–	186	214	400

### Attributable risk of hemorrhagic and ischemic stroke death due to high sodium intake

The PAFs for hemorrhagic and ischemic stroke deaths due to high sodium intake in Zhenjiang City from 2010 to 2021 are detailed in Table 2. During the study period, the attributable risk of high sodium intake-induced hemorrhagic and ischemic stroke deaths showed a downward trend among both males and females. The PAFs for stroke deaths due to high sodium intake varied between 12.0% and 19.3% across different years and gender groups, with male populations consistently showing lower PAFs than female populations.

**Table 2:** PAFs of hemorrhagic and ischemic stroke deaths due to high sodium intake in Zhenjiang city from 2010 to 2021.

Year	Hemorrhagic stroke PAF%			Ischemic stroke PAF%			Both PAF%		
	Male	Female	total	Male	Female	total	Male	Female	total
2010	15.6	16.8	16.2	17.5	19.3	18.5	16.1	17.5	16.8
2011	15.2	17.7	16.3	16.8	18.6	17.7	15.7	18.0	16.8
2012	14.3	17.1	15.7	16.0	18.2	17.2	14.9	17.6	16.3
2013	14.1	16.0	15.1	15.5	17.3	16.4	14.7	16.6	15.7
2014	14.4	17.3	15.8	14.8	17.8	16.4	14.6	17.6	16.1
2015	13.6	17.8	15.6	15.2	19.0	17.2	14.4	18.4	16.4
2016	12.8	16.9	14.8	14.0	18.4	16.4	13.4	17.8	15.7
2017	12.2	16.1	14.1	13.5	16.6	15.2	12.9	16.4	14.7
2018	12.3	16.1	14.1	13.9	17.8	16.0	13.1	17.0	15.1
2019	12.2	15.6	13.8	14.2	18.0	16.2	13.1	16.9	15.0
2020	12.0	14.9	13.4	15.5	18.1	16.8	13.7	16.5	15.1
2021	12.2	14.1	13.1	13.9	15.7	14.9	13.0	15.0	14.0

### Trends in mortality from hemorrhagic and ischemic stroke attributable to high sodium intake

The mortality trend of hemorrhagic and ischemic stroke deaths caused by high sodium intake is detailed in Table 3. From 2010 to 2021, the number of deaths attributed to high sodium intake in Zhenjiang City decreased from 426 to 390. Although the decline in deaths was relatively small, the ASMR showed a significant downward trend, decreasing from 58.33/100,000 to 23.11/100,000, with an AAPC (average annual percentage change) of −8.10% (95% CI (confidence interval): −12.00% to −3.90%), and the decline was slightly more pronounced in males than females. The number of deaths caused by hemorrhagic stroke due to high sodium intake decreased from 303 to 181, while the ASMR dropped from 40.01/100,000 to 10.97/100,000, with an AAPC of −11.10% (95% CI: −13.30% to −8.90%), and the decline in males was slightly less than that in females. The ASMR for ischemic stroke caused by high sodium intake showed an initial upward trend followed by a decline, the ASMR peaked in 2013 at 34.33/100,000, with AAPC of 22.80% (95% CI: 2.2%−47.5%) and −12.30% (−14.80% to −9.70%). The rising trend in ASMR for ischemic stroke among males showed no statistically significant difference between 2010 and 2013 (*p* > 0.05), while the ASMR decline amplitude in males and females was almost equal from 2013 to 2021. Notably, the ASMR for hemorrhagic and ischemic stroke attributable to high sodium intake remained higher in females than in males across all years.

**Table 3:** ASMR and its trend of hemorrhagic and ischemic stroke caused by high sodium intake in Zhenjiang city from 2010 to 2021.

Gender	Total			Hemorrhagic stroke			Ischemic stroke				
	2010 ASMR (95%UI)	2021 ASMR (95%UI)	AAPC% (95%CI)	2010 ASMR (95%UI)	2021 ASMR (95%UI)	AAPC% (95%CI)	2010 ASMR (95%UI)	2013 ASMR (95%UI)	2021 ASMR (95%UI)	AAPC% (2010–2013) (95%CI)	AAPC% (2013–2021) (95%CI)
Both	58.33	23.11	−8.10	40.01	10.97	−11.10	18.54	34.33	12.07	22.8	−12.30
	52.07 64.59	20.81 25.41	−12.00 −3.90	34.82 45.20	9.37 12.57	−13.30 −8.90	15.07 22.01	29.57 39.09	10.39 13.75	2.20 47.50	−14.80 −9.70
Male	56.89	20.62	−8.80	40.54	10.22	−11.80	17.56	27.52	9.94	16.2	−12.00
	48.29 65.49	17.56 23.68	−13.40 −4.00	33.23 47.85	7.98 12.46	−14.10 −9.40	12.82 22.30	21.65 33.39	7.81 12.07	−6.60 44.50	−15.00 −8.80
Female	61.12	24.61	−7.90	48.63	11.13	−12.50	20.84	39.42	14.18	23.7	−12.00
	52.09 70.15	21.22 28.00	−11.20 −4.50	41.12 56.14	8.91 13.35	−14.60 −10.40	15.63 26.05	33.02 45.82	11.65 16.72	1.40 50.90	−14.80 9.20

### Mortality from hemorrhagic and ischemic stroke attributable to high sodium intake

**Figure 2:**
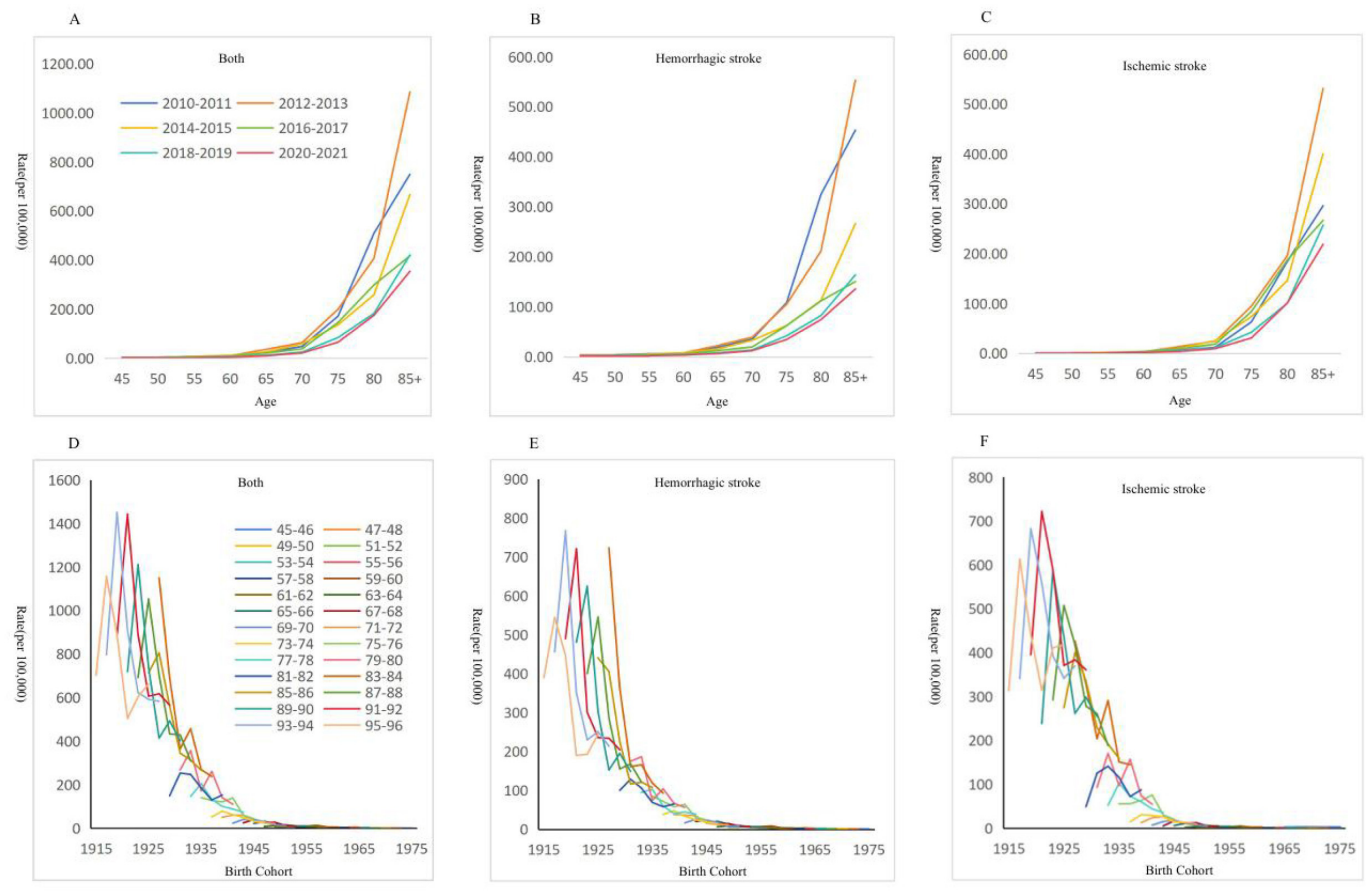
Age and Cohort specific mortality rates (per 100,000 population) of hemorrhagic and ischemic stroke caused by high sodium intake in Zhenjiang City from 2010 to 2021. **(A–C)** The survey years were divided into consecutive 2-year periods: 2010–2011 period, 2012–2013 period, 2014–2015 period, 2016–2017 period, 2018–2019 period, and 2020–2021 period, with the median value taken from the smaller year. **(D–F)** Data on mortality rates from hemorrhagic and ischemic stroke caused by high sodium intake were categorized into 31 consecutive birth groups: 1914–1915 group, 1916–1917 group, 1918–1919 group, 1920–1921 group, 1922–1923 group, 1924–1925 group, 1926–1927 group, 1928–1929 group, 1930–1931 group, 1932–1933 group, 1934–1935 group, 1936–1937 group, 1938–1939 group, 1940–1941 group, 1942–1943 group, 1944–1945 group, 1946–1947 group, 1948–1949 group, 1950–1951 group, 1952–1953 group, 1954–1955 group, 1956–1957 group, 1958–1959 group, 1960–1961 group, 1962–1963 group, 1964–1965 group, 1966–1967 group, 1968–1969 group, 1970–1971 group, 1972–1973 group, and 1974–1975 group, with the median value taken from the smaller year.

Mortality rates attributable to high sodium intake-induced hemorrhagic and ischemic stroke increased with age from 2010 to 2021. Notably, the mortality trends for both types of strokes showed consistent patterns across all periods (Figures 2A–C). Within each age group, the mortality rate from hemorrhagic stroke attributable to high sodium intake demonstrated a significant overall decline between 2010–2011 and 2020–2021 (Figure 2B). Conversely, the mortality rate from ischemic stroke attributable to high sodium intake exhibited an upward trend in most age groups during 2010–2011 to 2012–2013, followed by a general downward trend thereafter (Figure 2C). Analysis of stroke mortality rates attributable to high sodium intake across different birth cohort groups revealed that both hemorrhagic and ischemic stroke mortality rates followed a pattern of initial rise followed by rapid decline (Figures 2D–F). This indicates that over extended periods, the risk of ischemic and hemorrhagic stroke mortality has been significantly reduced across birth groups. Detailed findings are presented in Figure 2.

### Net drift and local drift in age groups

**Figure 3:**
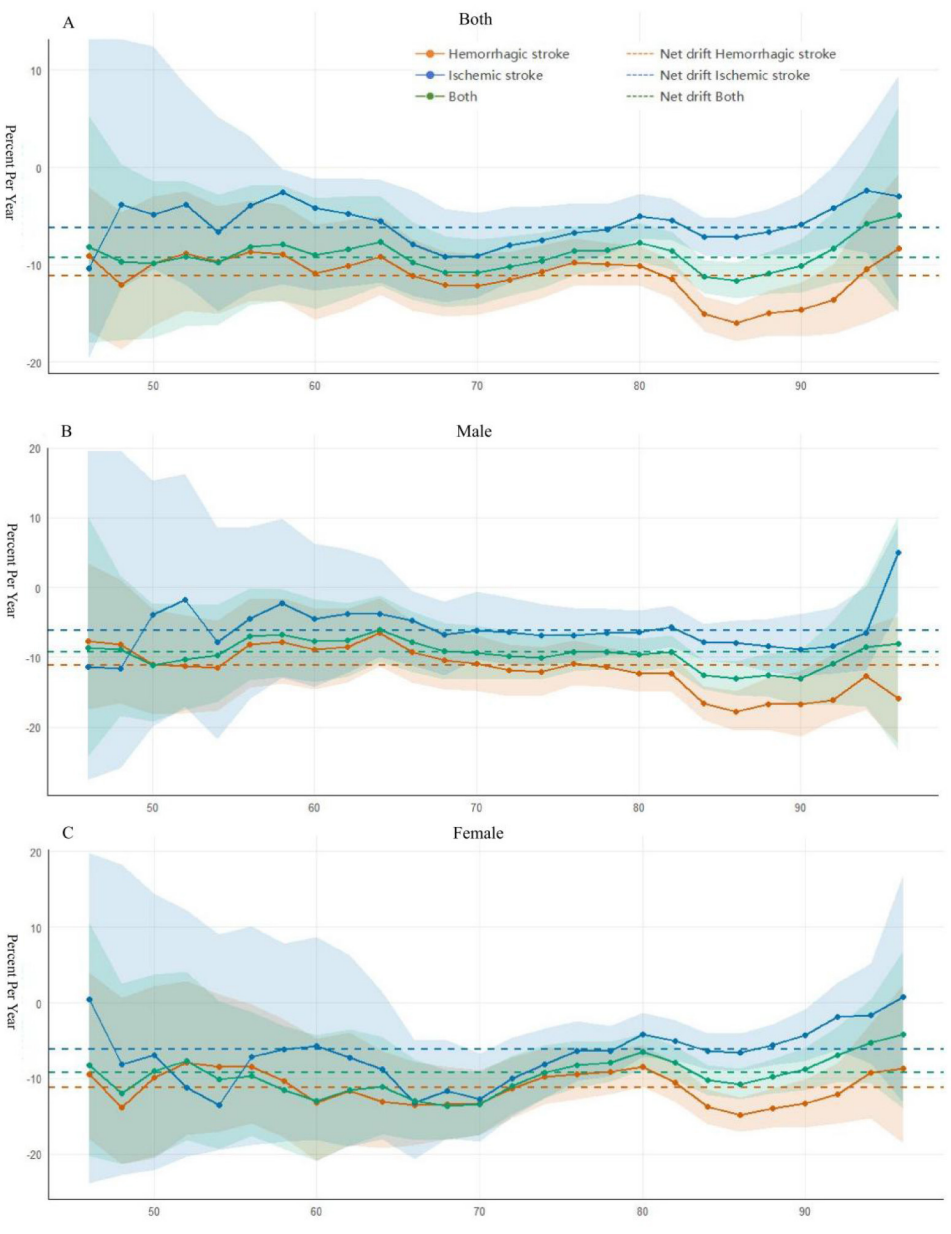
Local drift with net drift values of hemorrhagic and ischemic stroke mortality caused by high sodium intake in Zhenjiang city from 2010 to 2021. **(A)** represents both sexes, **(B)** represents males and **(C)** represents females. The dots and shaded areas represent the percentage and their corresponding 95% confidence intervals.

The net drift represents the estimated annual average percentage change (AAPC) in mortality rates over the entire study period, while the local drift reflects the AAPC of mortality rates across different age groups (Figure 3). From 2010 to 2021, the overall net drift for hemorrhagic and ischemic stroke mortality due to high sodium intake showed a downward trend (−9.25%,95% CI: −10.76% −7.72%), with the decline in hemorrhagic stroke mortality (−11.16%,95% CI: −12.49% −9.81%) being significantly greater than that of ischemic stroke mortality (−6.19%,95% CI: −8.57% −3.75%). The overall net drift for men and women were −11.42% (95% CI: −13.07% −9.74%) and −11.38% (95% CI: −13.39% −9.31%) in hemorrhagic stroke mortality, respectively, indicating equal mortality reduction amplitude. For ischemic stroke mortality, the overall net drift values were −5.92% (95% CI: −8.79% −2.95%) and −7.77% (95% CI: −11.51% −3.88%) in men and women, respectively, with women showing a more pronounced decrease amplitude in mortality rates compared to men.

In all age groups, the local drift values for hemorrhagic stroke mortality caused by high sodium intake remained below 0, indicating a significant downward trend in mortality rates. The most substantial decreases were observed in both males and females within the 85–86 age group, with local drift values of −17.72% (95% CI: −20.53 −14.81) and −14.83% (95% CI: −17.10 −12.50), respectively. The local drift values for ischemic stroke mortality caused by high sodium intake remained below 0, however, statistically significant declines were observed between 65 and 90 age in males and females. Males showed the greatest decrease in the 89–90 age group, while females experienced the most notable reduction in the 65–66 age group, with local drift values of −8.90% (95% CI: −12.73 −3.81) and −13.15% (95% CI: −20.67 −4.93), respectively. Hemorrhagic and ischemic stroke mortality maintained relatively stable levels before age 80, followed by initial decline and subsequent increase after 80 years of age.

### The APC effect of hemorrhagic and ischemic stroke mortality due to high sodium intake

**Figure 4:**
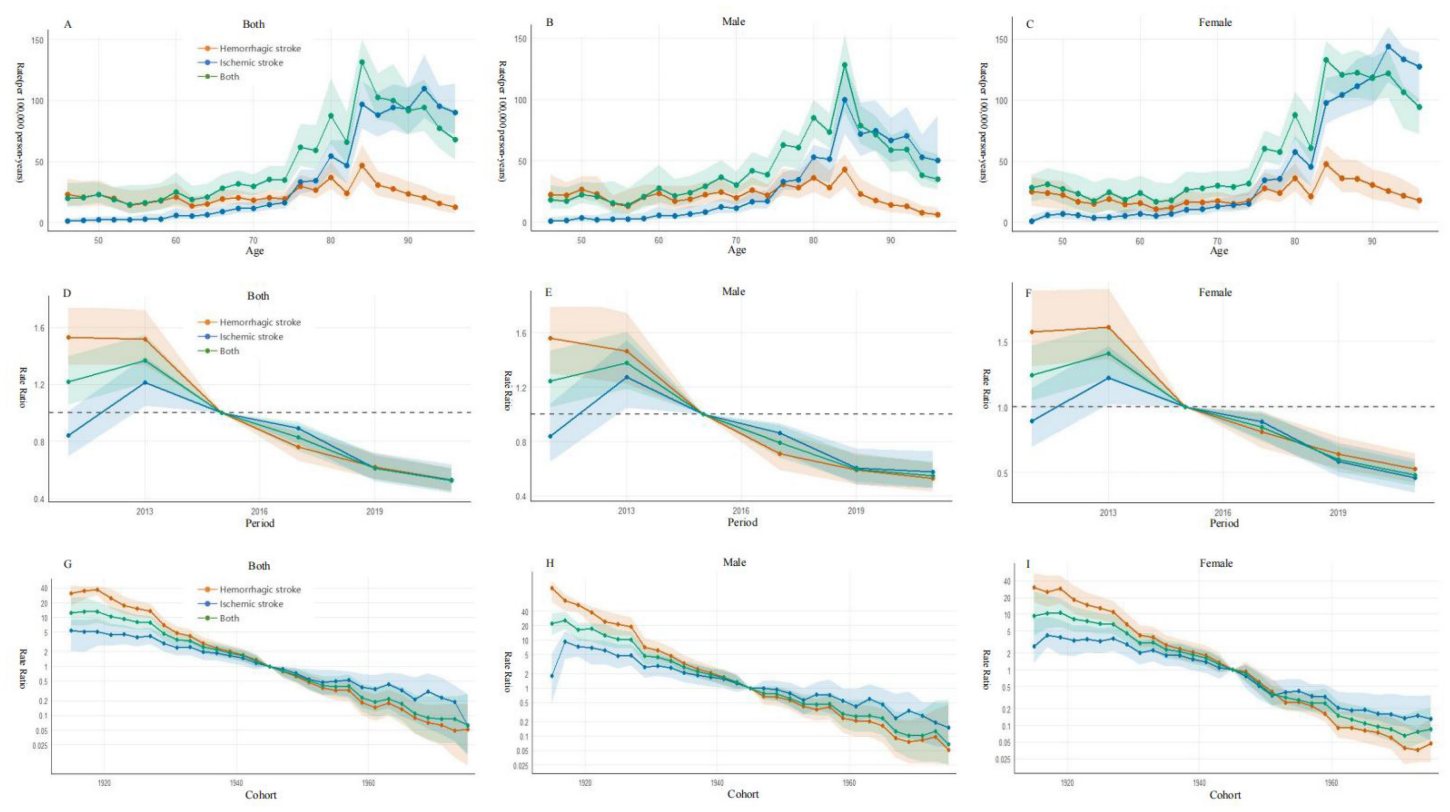
APC analysis results of hemorrhagic and ischemic stroke mortality caused by high sodium intake in Zhenjiang city from 2010 to 2021. **(A–C)** Longitudinal age curves of hemorrhagic and ischemic stroke mortality rates (per 100,000 population) caused by high sodium intake. **(A)** represents both genders, **(B)** represents males, and **(C)** represents females. **(D–F)** RR and corresponding 95% CI for each period compared with the reference period (2014–2015), adjusted for age and nonlinear cohort effects. **(D)** represents both genders, **(E)** represents males, and **(F)** represents females. **(G–I)** RR and corresponding 95% CI for each cohort group compared with the reference cohort group (1944–1945), adjusted for age and nonlinear period effects. **(G)** represents both genders, **(H)** represents males, and **(I)** represents females. Dots and shaded areas indicate mortality rates or RR along with their corresponding 95% CI.

The impact of age on mortality rates from hemorrhagic and ischemic stroke caused by high sodium intake shows an initial upward trend followed by a decline (Figures 4A–C). The increase accelerates in the 73–84 age group, while a downward trend emerges after 85 years old, with this pattern being more pronounced in males. After 85 years old, the mortality rate from hemorrhagic stroke due to high sodium intake declines rapidly, whereas that from ischemic stroke remains at a fixed level. This gender difference manifests as: after 85 years old, both hemorrhagic and ischemic stroke mortality rates show rapid decreases in males, while hemorrhagic stroke mortality rate decreased and ischemic stroke mortality rate increased in females. Trend figures further reveal that before age 73, hemorrhagic stroke mortality rates exceed those from ischemic stroke, but after age 73, ischemic stroke mortality surpasses hemorrhagic stroke, this phenomenon observed both in males and females.

The period effect of hemorrhagic and ischemic stroke mortality caused by high sodium intake showed a gradual increase (2010–2013) followed by a rapid decline (2013–2021), indicating effective control of high sodium intake during the study period, and the stroke mortality risk was almost equivalent in men and women (Figures 4D–F). The mortality risk from hemorrhagic stroke caused by high sodium intake maintained a downward trend, with a more pronounced decrease after 2013, meanwhile, the mortality risk from ischemic stroke began declining after 2013. Before 2017, the improvement in mortality risk from hemorrhagic stroke due to high sodium intake was higher than that for ischemic stroke. From 2018 to 2019, the improvement in hemorrhagic stroke mortality risk was lower than that for ischemic stroke, while from 2020 to 2021, the improvement trends were largely consistent between the two conditions and showed similar patterns in both males and females. After 2013, the improvement in ischemic stroke mortality risk was higher in women than men during 2014–2017, but reversed to male dominance in 2018–2021.

The risk of mortality from hemorrhagic and ischemic stroke caused by high sodium intake showed an overall downward trend in the study cohort group, with more pronounced declines observed in hemorrhagic stroke cases and among male populations (Figures 4G–I). This indicates that cohort effects positively influenced mortality rates from hemorrhagic and ischemic stroke caused by high sodium intake, particularly demonstrating stronger efficacy in male hemorrhagic stroke cases. However, an unfavorable trend emerged in cohort groups born after 1968, which became more evident in female populations. Although cohort effects demonstrated lower mortality reduction for ischemic stroke compared to hemorrhagic stroke, they exhibited a more favorable trend in cohort groups born after 1962, with this positive correlation being more pronounced in males.

## Discussion

Nestled along the Yangtze River in southern Jiangsu Province, Zhenjiang serves as a vital transportation hub connecting the Yangtze River and the Beijing-Hangzhou Grand Canal, making it a key city in the Yangtze River Delta Metropolitan Area. The locals have developed a unique breakfast tradition featuring wok-lid noodles, whose broth ingredient is primarily made from simmered soy sauce-a staple that plays a significant role in their high-sodium diet.

This study, the first global report of its kind, combines authoritative GBD data with local Zhenjiang monitoring to assess high sodium intake-related hemorrhagic and ischemic stroke mortality risk in Zhenjiang City, a city along China's lower Yangtze River, from 2010 to 2021. It provides city-level PAFs for high sodium intake-related deaths from hemorrhagic and ischemic stroke. While Tianjin City in China had previously reported PAFs for high sodium intake-related stroke mortality in northern China (2015 data) (Xue et al., 2023), this study offers a dynamic annual assessment from 2010 to 2021, demonstrating a clear downward trend in PAFs. Notably, Zhenjiang's daily dietary average sodium intake is lower than Tianjin's, consistent with the common knowledge that northern China consumes more salt than southern regions (Anderson et al., 2010). However, Zhenjiang's higher PAFs for high sodium intake-related stroke mortality suggest dietary sodium intake contributes a greater proportion in overall stroke risk factors compared to Tianjin, warranting heightened public attention.

Using the APC analysis model we systematically evaluated the independent effects of age, period and cohort factors on hemorrhagic and ischemic stroke mortality. From 2010 to 2021, the ASMR for hemorrhagic and ischemic stroke caused by high sodium intake in Zhenjiang residents showed a downward trend, indicating that the salt reduction intervention policies implemented since 2010 have significantly suppressed stroke mortality. The analysis revealed that the ASMR for stroke caused by high sodium intake in women consistently exceeded that in men, which may be related to the higher proportion of stroke deaths, the higher mortality ranking, and the greater disease burden in women compared to men (Heron, 2021; Virani et al., 2021). Additionally, it was associated with women's higher PAFs for high sodium intake-related stroke mortality across all age groups, meaning women in Zhenjiang experienced more severe SBP elevation due to high sodium intake than men. This demonstrates that high sodium intake has a greater impact on women's blood pressure than men, thereby increasing the risk of stroke (particularly ischemic stroke) (Ji et al., 2020). Relevant studies indicate that women exhibit higher sodium sensitivity (Balafa and Kalaitzidis, 2021; Liu et al., 2020) due to differences in hormonal regulation (Pechère-Bertschi and Burnier, 2004) and renal sodium processing function (Frame and Wainford, 2017) compared to men. Related studies (GBD 2016 Lifetime Risk of Stroke Collaborators et al., 2018) also reported that women after age 25 have a stroke risk of 1/4, indicating their lifetime stroke risk is higher than that of men. The risk reduction in ischemic stroke mortality from high sodium intake among women was more pronounced than in men from 2014 to 2017, but declined below men levels from 2018 to 2021. This aligns with cohort analysis findings indicating more favorable trends in reduction of ischemic stroke mortality among males born after 1962. While women show higher net drift values for high sodium intake-related ischemic stroke mortality compared to men, recent positive improvements have emerged in males under 60 years old. These findings suggest that although the risk of stroke from high sodium intake has been partially controlled, primary stroke prevention strategies require reinforcement (Feigin et al., 2020, 2024), targeted adjustments should focus on high-risk population and key risk factors such as sodium intake. Although studies report gender differences in cerebral hemorrhage burden caused by estrogen's cardiovascular protective effects (Reeves et al., 2008; Ryu et al., 2023), our research reveals a strong correlation between high sodium intake and women's stroke risk (particularly ischemic stroke). This provides substantial theoretical support for developing region-specific stroke prevention and treatment guidelines prioritizing women in our city and broader areas (Rexrode et al., 2022).

Age stratified analysis reveals that mortality rates from hemorrhagic and ischemic stroke caused by high sodium intake rise sharply with age, particularly after 73 years old when this upward trend becomes more pronounced. This phenomenon may be linked to the aging population being recognized as a primary risk group for cardiovascular diseases (CVD) (Wang et al., 2017). Studies have shown that reducing dietary sodium intake significantly lowers blood pressure in most elderly individuals (Huang et al., 2020; Gupta et al., 2023), indicating that older adults are more susceptible to stroke-related cerebrovascular events due to increased vulnerability to hypertension caused by excessive sodium consumption. The occurrence of this phenomenon may be related to diminished compensatory capacity in elderly patients' bodies to counteract elevated blood pressure resulting from high sodium intake, as their physical functions gradually decline.

From 2010 to 2021, the net drift in mortality rates from hemorrhagic and ischemic stroke attributable to high sodium intake showed a downward trend. Notably, the decline amplitude in hemorrhagic stroke mortality outpaced that of ischemic stroke. This phenomenon can be explained by period effect analysis result: The risk of hemorrhagic stroke mortality due to high sodium intake maintained a consistent downward trend, while ischemic stroke mortality risk only began decreasing after 2013. Furthermore, before 2017, the improvement in hemorrhagic stroke mortality risk due to high sodium intake consistently exceeded that of ischemic stroke. Relevant studies have reported that high sodium intake has a greater impact on hemorrhagic stroke than ischemic stroke (Li et al., 2018; Judge et al., 2021), indicating that when this risk factor is effectively controlled, the population's benefit from hemorrhagic stroke prevention will inevitably surpass that of ischemic stroke.

During the study period, mortality rates of hemorrhagic and ischemic stroke attributable to high sodium intake across all age groups showed a trend of initial increase followed by decline. Combined with period effect analysis results, this indicates that the risk of stroke mortality has significantly improved in recent years. To address challenges of chronic non-communicable diseases (NCDs) such as stroke, government authorities have implemented policies including the “Medium-to-Long-Term Plan for the Prevention and Treatment of Chronic Disease in Jiangsu (2018–2025)” (Jiangsu Provincial People's Government, 2018) and “Healthy Jiangsu 2030” (Jiangsu Commission of Health, 2017). Notably, Zhenjiang City has systematically implemented the “Healthy Zhenjiang” action plan since 2010, dynamically adjusting disease prevention and health promotion measures to advance population health interventions. In recent years, under the support of the “Healthy Zhenjiang” Implementation Plan (2020–2023) (Zhenjiang City People's Government, 2020), efforts to intervene in dietary risk factors like high sodium, fat, and sugar intake have been intensified. The declining trend observed during the study period suggests that health benefits from these interventions are beginning to manifest. However, different from the local drift value for hemorrhagic stroke mortality remains below zero, the local drift value for ischemic stroke only falls below zero among individuals aged 65–90. This indicates that the decline in ischemic stroke mortality has not offset the effects of population growth and aging. Therefore, it is imperative to strengthen health interventions related to high sodium intake and ischemic stroke in both middle-aged and elderly populations.

Longitudinal age effect analysis reveals that high sodium intake causes a rapid increase in mortality rates from hemorrhagic and ischemic stroke among individuals aged 73–84, followed by a sharp decline after age 85. This trend may be linked to population aging and extended life expectancy, as aging elevates stroke risk (Li et al., 2025). However, elderly individuals exhibit suppressed prevalence of stroke risk factors due to their stable lifestyles, higher adherence to health interventions, and survival effects. Notably, ischemic stroke mortality rates in females showed an upward trend after age 85, which could be attributed to women's longer life expectancy and greater representation in the older age group.

The cohort analysis revealed that mortality risk for hemorrhagic and ischemic stroke caused by high sodium intake showed a downward trend, with this decline being more pronounced among males with hemorrhagic stroke. However, unfavorable trends emerged in post-1968 cohort groups, particularly evident in females. Although the cohort effect demonstrated greater impact on hemorrhagic stroke mortality than ischemic stroke, it should be noted that favorable trends on decline of ischemic stroke mortality became more apparent in male cohorts after 1962. These suggest that rapid economic growth and urbanization have led to significant changes in residents' lifestyles and dietary patterns, with unhealthy lifestyles and health risk factors becoming increasingly prevalent. Concurrently, improvements in population nutritional status, education levels, and healthcare service quality (Wang et al., 2017) have promoted positive developments in stroke risk reduction and salt-reduction awareness. These resulted in significant differences among various cohort groups regarding the improvement in stroke-related mortality attributed to high sodium intake. Nevertheless, certain differences in the period effects among specific cohort groups may also exist.

This study employed the APC model analysis tool to conduct a time trend analysis. It provides a combination of parameters for the time trends, which can be statistically uniquely estimated (e.g., net drift, local drift, etc.), shifting the focus from absolute statistical effects to reliable changes and comparative trends. This provides scientific insights for regional disease burden analysis, while offering evidence-based support for identifying priority populations in hemorrhagic and ischemic stroke prevention within the region, strengthening interventions targeting high sodium intake, and optimizing the utilization of social prevention resources. Although the model mitigates the uncertainty issue caused by indicator collinearity by estimating a recognizable function, its analysis is based on the Poisson distribution and inadequately addresses the problem of excessive dispersion. This may increase the probability of false-positive conclusions, thereby compromising the validity of the findings in this study. Additionally, the impact of sodium intake on stroke mortality was indirectly derived through its correlation with SBP, the reference for the RR of elevated SBP due to high sodium intake was based on global GBD data rather than Zhenjiang's local data, and while the calculation method is rooted in scientific theory, it may still exhibit deviations from actual conditions. During data processing, we substituted the missing sodium intake and SBP data for specific years with the average value from the adjacent two years, although this alternative approach ensures data continuity and provides necessary control over the variability of missing data to some extent, but this reduced the accuracy of research findings. Considering the continuity and comparability of the work, particularly factors such as technical requirements and funding, the method for measuring sodium intake in our study is not the gold standard of 24-h urine sodium testing (Chiang et al., 2024; Campbell et al., 2019). Although confounding factors such as gender and age structure were considered in the analysis process, indicators like body mass index (BMI) and comorbidities of chronic diseases were not included in the study, the attributable risks may be distorted. Overall, this study constitutes an ecological investigation focusing on the temporal trends of attributable risk. While the identified research insights provide a scientific basis for improving current stroke prevention and control measures, they do not establish robust causal relationships. Future research should prioritize prospective cohort studies to evaluate the implementation effects of specific stroke prevention policies—particularly those emphasizing salt reduction—and thereby provide solid evidence for developing and implementing high-efficiency stroke prevention strategies in community populations.

## Conclusion

In Zhenjiang City, the mortality rates from hemorrhagic stroke and ischemic stroke caused by high sodium intake have significantly decreased across all age groups. While women and the elderly show higher attributable mortality risks, their improvement in mortality rates remains notable. Although the reduction in hemorrhagic stroke mortality caused by high sodium intake has outperformed that of ischemic stroke, females born after 1968 have experienced unfavorable trends in hemorrhagic stroke mortality, whereas males born after 1962 have shown favorable trends in ischemic stroke mortality. To curb the impact of high sodium intake on stroke mortality, targeted health policies and measures should be continuously implemented, along with intensified community salt reduction initiatives.

## Data Availability

Data cannot be shared publicly, because data from this study may contain potentially or sensitive patient information. While the relevant information has been listed in the Supplementary Table. Requests to access the datasets should be directed to Xiaoyong Gu, lanqigxy@163.com.
