# Supplementary material for: Attributable risk and time trend in hemorrhagic and ischemic stroke mortality due to high sodium intake in Zhenjiang City from 2010 to 2021: an Age-Period-Cohort (APC) analysis
Source: Front Stroke. 2026 Jun 25;5:1722772. doi: 10.3389/fstro.2026.1722772 (PMC13345931; doi:10.3389/fstro.2026.1722772)
Supplement: Supplementary file 1 [file Supplementary_File_1.docx]

Supplementary Material 1. The general log-linear form and calculation method for the APC model.

The general log-linear form of the APC model is expressed as the follow (18):

$\rho=\alpha_{a}+\beta_{p}+\gamma_{c}$ (1)

Here, *ρ* represents the expected incidence rate, $\alpha_{a}\text{,}\beta_{p} and \gamma_{c}$ respectively represent age, period and cohort effects. The age-period model (2) and age-cohort model (3) are as follows:

$\rho_{ap}=\mu+(\alpha_{L}-\gamma_{L})(a-\bar{a})+(\pi_{L}+\gamma_{L})(p-\bar{p})+\tilde{\alpha_{a}}+\tilde{\pi_{p}}+\tilde{\gamma_{c}}$ (2)

$\rho_{ac}=\mu+(\alpha_{L}+\pi_{L})(a-\bar{a})+(\pi_{L}+\gamma_{L})(c-\bar{c})+\tilde{\alpha_{a}}+\tilde{\pi_{p}}+\tilde{\gamma_{c}}$ (3)

Where $\alpha_{L}-\gamma_{L}$ represents the the cross-sectional trend, $\alpha_{L}+\pi_{L}$ represents the longitudinal age trend, $\pi_{L}+\gamma_{L}$ represents net drift, $\tilde{\alpha_{a}}$, $\tilde{\pi_{p}}$ and $\tilde{\gamma_{c}}$ represent age, period and cohort bias, respectively.

The analysis utilized the APC model analysis tool developed by the Biostatistics Division of the National Institutes of Health (NIH) (19). In this model, collected data were transformed into continuous 2-year age groups and 2-year time intervals. Demographic and mortality data were categorized into 26 age groups: 45-46 years group, 47-48 years group, 49-50 years group, 51-52 years group, 53-54 years group, 55-56 years group, 57-58 years group, 59-60 years group, 61-62 years group, 63-64 years group, 65-66 years group, 67-68 years group, 69-70 years group, 71-72 years group, 73-74 years group, 75-76 years group, 77-78 years group, 79-80 years group, 81-82 years group, 83-84 years group, 85-86 years group, 87-88 years group, 89-90 years group, 91-92 years group, 93-94 years group, and 95-96 years group. As cases of stroke-related deaths caused by high sodium intake in individuals under 45 years are rare (about 1.02% in our study), this study excluded data from this age group.

The period was divided into six consecutive two-year periods: 2010-2011period, 2012-2013period, 2014-2015period, 2016-2017period, 2018-2019period, and 2020-2021period. Based on the relationships between age, periods, and cohorts, 31 consecutive birth cohorts were generated by subtracting the death years from the periods of death as follows: 1914-1915 group, 1916-1917 group, 1918-1919 group, 1920-1921 group, 1922-1923 group, 1924-1925 group, 1926-1927 group, 1928-1929 group, 1930-1931 group, 1932-1933 group, 1934-1935 group, 1936-1937 group, 1938-1939 group, 1940-1941 group, 1942-1943 group, 1944-1945 group, 1946-1947 group, 1948-1949 group, 1950-1951 group, 1952-1953 group, 1954-1955 group, 1956-1957 group, 1958-1959 group, 1960-1961 group, 1962-1963 group, 1964-1965 group, 1966-1967 group, 1968-1969 group, 1970-1971 group, 1972-1973 group, 1974-1975 group. All APC analyses were performed with the initial age group, period or birth cohort as representative groups.
