# Supplementary material for: Attributable risk and time trend in hemorrhagic and ischemic stroke mortality due to high sodium intake in Zhenjiang City from 2010 to 2021: an Age-Period-Cohort (APC) analysis
Source: Front Stroke. 2026 Jun 25;5:1722772. doi: 10.3389/fstro.2026.1722772 (PMC13345931; doi:10.3389/fstro.2026.1722772)
Supplement: Supplementary file 2 [file Table_1.docx]

Table S1 Distribution of systolic blood pressure(SBP) among different groups from 2011 to 2020 (mmHg)

| **Age group** | **2011** | | | **2012** | | | **2013** | | | **2014** | | | **2015** | | | **2016** | | | **2017** | | | **2020** | | | **total** | | |
| --- | --- | --- | --- | --- | --- | --- | --- | --- | --- | --- | --- | --- | --- | --- | --- | --- | --- | --- | --- | --- | --- | --- | --- | --- | --- | --- | --- |
|  | **both** | **male** | **female** | **both** | **male** | **female** | **both** | **male** | **female** | **both** | **male** | **female** | **both** | **male** | **female** | **both** | **male** | **female** | **both** | **male** | **female** | **both** | **male** | **female** | **both** | **male** | **female** |
| 45-46 | 125.9 | 129.5 | 122.9 | 127.7 | 132.2 | 123.6 | 127.2 | 128.2 | 126.1 | 127.0 | 129.7 | 123.7 | 128.5 | 129.7 | 127.2 | 126.9 | 131.0 | 122.7 | 129.4 | 131.5 | 126.9 | 128.4 | 133.2 | 123.4 | 127.6 | 130.5 | 124.5 |
| 47-48 | 126.6 | 127.3 | 126.1 | 125.9 | 125.6 | 126.1 | 130.0 | 129.0 | 131.1 | 128.5 | 132.0 | 124.7 | 127.6 | 130.4 | 124.7 | 127.0 | 131.6 | 121.6 | 129.2 | 131.3 | 126.5 | 129.6 | 133.7 | 125.2 | 128.1 | 130.3 | 125.9 |
| 49-50 | 130.5 | 130.0 | 130.8 | 128.3 | 132.4 | 125.4 | 131.0 | 130.9 | 131.0 | 129.4 | 130.5 | 128.3 | 129.1 | 130.8 | 127.4 | 130.1 | 131.7 | 128.6 | 128.5 | 129.2 | 128.0 | 130.5 | 132.6 | 128.6 | 129.7 | 131.1 | 128.5 |
| 51-52 | 131.6 | 131.0 | 132.0 | 130.3 | 132.6 | 128.6 | 129.8 | 131.6 | 128.4 | 131.6 | 132.8 | 130.1 | 130.9 | 132.7 | 129.1 | 131.1 | 132.3 | 129.9 | 129.9 | 131.9 | 127.7 | 131.6 | 132.9 | 130.6 | 131.0 | 132.4 | 129.7 |
| 53-54 | 131.2 | 131.4 | 131.0 | 131.9 | 133.1 | 130.8 | 131.1 | 132.7 | 129.7 | 132.2 | 131.8 | 132.5 | 129.3 | 130.2 | 128.5 | 131.0 | 133.5 | 128.3 | 133.2 | 133.2 | 133.1 | 133.0 | 136.5 | 130.2 | 131.7 | 133.1 | 130.5 |
| 55-56 | 130.6 | 135.1 | 126.5 | 131.0 | 132.1 | 130.1 | 133.6 | 137.9 | 129.8 | 130.6 | 130.4 | 130.8 | 131.8 | 134.5 | 129.0 | 133.0 | 136.3 | 129.5 | 131.0 | 132.1 | 129.8 | 134.9 | 136.7 | 133.7 | 132.3 | 134.6 | 130.2 |
| 57-58 | 132.4 | 131.8 | 132.8 | 133.6 | 136.5 | 131.6 | 131.8 | 131.0 | 132.6 | 129.7 | 130.4 | 129.1 | 132.4 | 133.9 | 130.1 | 132.1 | 134.9 | 129.6 | 133.2 | 132.3 | 134.2 | 135.3 | 135.7 | 134.9 | 132.7 | 133.4 | 132.2 |
| 59-60 | 134.5 | 134.5 | 134.5 | 134.3 | 137.1 | 132.4 | 134.5 | 134.4 | 134.7 | 132.9 | 132.8 | 133.1 | 131.5 | 132.1 | 130.9 | 134.2 | 135.3 | 133.1 | 134.1 | 134.8 | 133.5 | 136.7 | 137.5 | 135.9 | 134.4 | 135.0 | 133.9 |
| 61-62 | 135.5 | 134.8 | 136.0 | 135.2 | 135.4 | 134.9 | 135.6 | 136.2 | 134.8 | 134.7 | 135.1 | 134.3 | 131.5 | 133.4 | 129.8 | 136.6 | 136.1 | 137.2 | 134.1 | 134.6 | 133.4 | 140.5 | 139.9 | 140.9 | 135.6 | 135.7 | 135.5 |
| 63-64 | 137.6 | 139.0 | 136.5 | 136.1 | 137.2 | 135.3 | 132.9 | 134.0 | 131.9 | 135.9 | 135.6 | 136.2 | 133.8 | 131.9 | 136.3 | 136.5 | 137.1 | 136.0 | 136.4 | 137.8 | 134.1 | 137.7 | 138.0 | 137.5 | 136.0 | 136.4 | 135.7 |
| 65-66 | 138.0 | 136.6 | 139.3 | 137.6 | 138.4 | 136.6 | 136.5 | 135.5 | 137.3 | 135.4 | 137.0 | 134.2 | 133.8 | 135.1 | 132.0 | 139.2 | 138.4 | 140.0 | 135.5 | 134.2 | 137.1 | 139.1 | 139.0 | 139.2 | 137.4 | 137.2 | 137.6 |
| 67-68 | 138.1 | 139.2 | 137.2 | 136.0 | 137.7 | 134.8 | 134.4 | 135.2 | 133.7 | 135.8 | 135.9 | 135.8 | 135.6 | 136.8 | 134.5 | 136.8 | 135.5 | 138.4 | 135.9 | 137.4 | 134.3 | 139.8 | 139.8 | 139.8 | 137.4 | 137.7 | 137.0 |
| 69-70 | 139.9 | 144.8 | 136.7 | 136.4 | 137.1 | 135.7 | 137.8 | 140.9 | 134.0 | 134.4 | 136.3 | 132.2 | 134.0 | 134.9 | 132.9 | 139.0 | 140.4 | 137.7 | 134.7 | 135.4 | 134.1 | 141.4 | 139.2 | 143.0 | 138.4 | 138.6 | 138.2 |
| 71-72 | 139.6 | 136.9 | 142.0 | 137.7 | 136.0 | 139.9 | 139.1 | 139.0 | 139.1 | 135.5 | 130.4 | 139.4 | 134.3 | 134.0 | 134.6 | 140.1 | 142.0 | 137.8 | 141.6 | 142.2 | 140.8 | 140.9 | 141.3 | 140.5 | 139.3 | 138.8 | 139.8 |
| 73-74 | 138.8 | 139.0 | 138.5 | 137.8 | 142.3 | 134.6 | 138.6 | 138.6 | 138.6 | 136.5 | 135.5 | 137.7 | 136.2 | 136.8 | 135.6 | 137.7 | 137.3 | 138.0 | 137.8 | 135.4 | 140.6 | 142.5 | 140.9 | 143.8 | 139.4 | 138.9 | 139.8 |
| 75-76 | 137.3 | 138.2 | 136.5 | 139.8 | 138.1 | 141.0 | 138.5 | 135.4 | 141.7 | 139.4 | 138.3 | 140.9 | 134.8 | 136.2 | 133.0 | 138.4 | 135.1 | 140.8 | 132.5 | 136.1 | 129.1 | 142.2 | 141.3 | 143.0 | 139.0 | 138.3 | 139.6 |
| 77-78 | 139.4 | 142.7 | 135.4 | 141.0 | 140.6 | 141.3 | 138.4 | 139.5 | 137.5 | 136.6 | 132.0 | 138.9 | 131.1 | 130.4 | 131.8 | 139.3 | 138.3 | 140.7 | 133.5 | 130.6 | 135.7 | 140.3 | 138.3 | 142.2 | 138.1 | 137.5 | 138.7 |
| 79-80 | 135.7 | 137.2 | 134.7 | 140.8 | 136.1 | 144.3 | 140.3 | 138.0 | 142.1 | 142.9 | 140.1 | 144.6 | 137.0 | 139.9 | 135.1 | 142.0 | 144.7 | 139.8 | 136.1 | 137.7 | 134.0 | 141.0 | 138.2 | 145.8 | 139.6 | 138.8 | 140.2 |
| 81-82 | 142.7 | 145.1 | 140.6 | 138.9 | 146.0 | 131.9 | 139.7 | 138.3 | 141.8 | 140.4 | 140.4 | 140.5 | 137.7 | 137.4 | 137.8 | 141.2 | 148.4 | 138.0 | 140.7 | 136.3 | 143.8 | 140.2 | 143.2 | 137.4 | 140.3 | 141.6 | 139.2 |
| 83-84 | 134.9 | 132.3 | 139.4 | 143.1 | 143.0 | 143.2 | 142.9 | 139.4 | 147.4 | 134.4 | 136.7 | 132.9 | 135.2 | 135.2 | 135.2 | 141.4 | 135.0 | 145.0 | 138.7 | 145.5 | 134.6 | 148.9 | 143.4 | 151.5 | 139.5 | 138.0 | 140.9 |
| ≥85 | 134.9 | 136.0 | 134.0 | 141.8 | 146.4 | 135.2 | 133.5 | 134.8 | 132.2 | 135.7 | 137.4 | 134.4 | 136.6 | 136.4 | 136.7 | 142.6 | 145.2 | 140.6 | 138.4 | 134.4 | 140.6 | 139.8 | 143.0 | 138.2 | 137.9 | 139.3 | 136.9 |
| total | 128.5 | 130.0 | 127.3 | 126.0 | 128.5 | 123.6 | 127.8 | 129.1 | 126.5 | 126.4 | 128.0 | 124.7 | 127.7 | 129.6 | 125.8 | 126.7 | 129.6 | 123.9 | 127.4 | 129.7 | 124.9 | 132.2 | 133.8 | 130.8 | 128.1 | 130.0 | 126.3 |

Table S2 Distribution of dietary sodium intake among different groups from 2012 to 2020 (mg/24h)

| **Age group** | **2012** | | | **2014** | | | **2015** | | | **2016** | | | **2017** | | | **2020** | | | **total** | | |
| --- | --- | --- | --- | --- | --- | --- | --- | --- | --- | --- | --- | --- | --- | --- | --- | --- | --- | --- | --- | --- | --- |
|  | **both** | **male** | **female** | **both** | **male** | **female** | **both** | **male** | **female** | **both** | **male** | **female** | **both** | **male** | **female** | **both** | **male** | **female** | **both** | **male** | **female** |
| 45-46 | 3816.0 | 4011.4 | 3635.2 | 3593.9 | 3746.8 | 3411.4 | 3501.1 | 3421.8 | 3589.5 | 3790.6 | 3809.0 | 3771.8 | 4022.4 | 3920.8 | 4149.0 | 3134.3 | 3254.3 | 3005.8 | 3627.6 | 3673.9 | 3577.0 |
| 47-48 | 4123.0 | 3824.9 | 4381.0 | 3710.1 | 3649.0 | 3774.7 | 3555.7 | 3709.7 | 3392.3 | 3939.5 | 3852.0 | 4041.5 | 3876.7 | 3970.4 | 3757.3 | 3374.8 | 3621.0 | 3115.9 | 3744.6 | 3765.9 | 3721.8 |
| 49-50 | 3825.4 | 4066.8 | 3651.8 | 3488.7 | 3355.9 | 3614.8 | 3538.6 | 3338.4 | 3746.1 | 3827.1 | 3601.2 | 4050.1 | 3796.1 | 3734.8 | 3846.8 | 3085.6 | 2976.5 | 3188.5 | 3559.6 | 3456.3 | 3653.9 |
| 51-52 | 3948.4 | 4186.9 | 3767.0 | 3596.4 | 3594.5 | 3598.7 | 3439.7 | 3523.4 | 3354.1 | 3724.5 | 3790.3 | 3660.2 | 3691.7 | 3707.1 | 3673.7 | 3290.4 | 3530.3 | 3086.9 | 3552.9 | 3655.0 | 3451.5 |
| 53-54 | 3817.9 | 3709.9 | 3918.3 | 3477.2 | 3708.2 | 3325.5 | 3553.1 | 3738.3 | 3387.0 | 3766.7 | 4049.3 | 3462.6 | 3829.7 | 3654.5 | 4040.4 | 3327.2 | 3413.9 | 3258.2 | 3606.7 | 3690.2 | 3530.1 |
| 55-56 | 3544.4 | 3597.3 | 3497.1 | 3592.6 | 3683.3 | 3519.5 | 3335.1 | 3437.3 | 3226.4 | 3711.7 | 3634.8 | 3794.5 | 3756.3 | 3797.9 | 3714.2 | 3284.0 | 3315.2 | 3262.7 | 3505.3 | 3557.7 | 3459.2 |
| 57-58 | 3690.8 | 3845.2 | 3583.7 | 3972.3 | 4359.6 | 3557.3 | 3547.3 | 3661.7 | 3383.2 | 3733.7 | 3532.8 | 3902.5 | 3774.2 | 3674.7 | 3885.2 | 3323.7 | 3289.9 | 3348.8 | 3628.0 | 3696.2 | 3565.2 |
| 59-60 | 3887.8 | 3840.9 | 3921.9 | 3724.2 | 3908.4 | 3520.1 | 3574.1 | 3581.4 | 3566.6 | 3920.8 | 3972.8 | 3864.4 | 4012.2 | 3842.9 | 4167.1 | 3379.5 | 3490.0 | 3286.0 | 3684.8 | 3731.3 | 3641.3 |
| 61-62 | 3477.0 | 3389.0 | 3618.2 | 3750.0 | 3800.5 | 3692.7 | 3552.4 | 3541.2 | 3562.3 | 4073.6 | 4349.9 | 3708.5 | 3847.4 | 3952.5 | 3693.8 | 2952.7 | 3095.7 | 2853.5 | 3585.7 | 3709.4 | 3450.6 |
| 63-64 | 3485.2 | 3331.5 | 3595.7 | 3782.3 | 3809.7 | 3750.1 | 4113.9 | 4156.6 | 4055.2 | 3877.7 | 3786.5 | 3967.3 | 4058.9 | 4121.9 | 3959.0 | 3537.5 | 3567.4 | 3511.6 | 3788.9 | 3814.9 | 3761.4 |
| 65-66 | 4234.4 | 3845.3 | 4718.5 | 3940.5 | 4283.4 | 3678.7 | 3788.4 | 3869.8 | 3680.0 | 3960.0 | 4036.6 | 3885.1 | 3792.2 | 3953.3 | 3593.9 | 3789.2 | 3956.4 | 3634.7 | 3881.8 | 3973.5 | 3788.4 |
| 67-68 | 4133.8 | 4173.4 | 4106.4 | 3983.3 | 3943.8 | 4026.8 | 4231.2 | 4536.1 | 3939.0 | 4043.5 | 4257.0 | 3780.3 | 4170.6 | 4117.2 | 4228.3 | 3514.8 | 3505.6 | 3522.8 | 3865.7 | 3935.0 | 3799.3 |
| 69-70 | 3867.2 | 3814.6 | 3918.4 | 4180.6 | 4061.4 | 4322.9 | 3907.0 | 4112.0 | 3645.5 | 3714.8 | 3787.3 | 3640.2 | 3818.7 | 3457.2 | 4137.2 | 3504.9 | 3466.9 | 3531.0 | 3719.7 | 3698.4 | 3738.3 |
| 71-72 | 4065.9 | 4112.7 | 4008.0 | 4148.0 | 4777.4 | 3671.1 | 4030.4 | 3604.4 | 4592.7 | 3772.5 | 3818.5 | 3716.8 | 3967.6 | 4006.9 | 3905.5 | 3911.6 | 3811.8 | 3996.5 | 3957.3 | 3935.8 | 3979.2 |
| 73-74 | 3779.9 | 3524.9 | 3963.1 | 4129.2 | 4106.0 | 4157.9 | 3787.0 | 4037.0 | 3537.0 | 3925.9 | 3448.2 | 4222.4 | 3684.1 | 3692.4 | 3674.9 | 3740.1 | 3703.0 | 3772.4 | 3813.0 | 3757.8 | 3862.3 |
| 75-76 | 4325.7 | 4161.5 | 4443.9 | 4698.5 | 4355.3 | 5176.0 | 4333.6 | 4455.9 | 4180.6 | 4174.4 | 4216.3 | 4143.4 | 3776.4 | 3514.8 | 4023.4 | 3638.0 | 3480.4 | 3788.1 | 4019.7 | 3894.6 | 4141.6 |
| 77-78 | 3928.5 | 4028.8 | 3858.7 | 4481.3 | 5328.5 | 4057.7 | 4125.7 | 3654.5 | 4678.8 | 3750.3 | 3450.8 | 4139.7 | 4163.3 | 4701.7 | 3759.4 | 3736.0 | 3726.8 | 3744.4 | 3948.0 | 3912.5 | 3979.3 |
| 79-80 | 5101.4 | 4633.4 | 5465.4 | 4732.2 | 4360.6 | 4955.2 | 4810.4 | 4259.9 | 5195.7 | 4939.6 | 4650.1 | 5174.8 | 4411.8 | 4363.5 | 4476.1 | 3815.2 | 3768.5 | 3895.6 | 4573.9 | 4234.1 | 4897.0 |
| 81-82 | 3404.6 | 3764.4 | 3044.8 | 4351.5 | 4167.5 | 4521.3 | 4169.9 | 3797.0 | 4475.1 | 3413.9 | 3140.3 | 3533.7 | 4202.6 | 4197.3 | 4206.3 | 3842.6 | 4551.5 | 3204.6 | 3924.0 | 4067.3 | 3813.0 |
| 83-84 | 5637.0 | 5095.7 | 6358.7 | 4680.0 | 6486.0 | 3530.8 | 4133.7 | 3703.5 | 4528.0 | 3955.1 | 2733.5 | 4653.1 | 4280.5 | 4012.3 | 4441.4 | 3548.9 | 3245.8 | 3686.6 | 4357.2 | 4345.0 | 4366.1 |
| ≥85 | 4586.7 | 4293.5 | 4986.5 | 4224.0 | 4656.8 | 3917.5 | 4410.7 | 4317.1 | 4488.6 | 4538.5 | 4173.7 | 4797.0 | 4076.2 | 4081.6 | 4073.0 | 3674.9 | 3394.2 | 3811.0 | 4229.2 | 4174.8 | 4268.1 |
| total | 3812.7 | 3784.0 | 3839.0 | 3631.4 | 3676.7 | 3584.7 | 3446.6 | 3478.8 | 3412.7 | 3679.4 | 3664.4 | 3694.2 | 3744.2 | 3701.4 | 3790.7 | 3349.4 | 3395.7 | 3309.6 | 3583.8 | 3597.6 | 3570.3 |

Table S3 Distribution of systolic blood pressure(SBP) increasing attributable to high sodium intake among different groups from 2012 to 2020 (mmHg)

| **Age group** | **2012** | | | **2014** | | | **2015** | | | **2016** | | | **2017** | | | **2020** | | | **total** | | |
| --- | --- | --- | --- | --- | --- | --- | --- | --- | --- | --- | --- | --- | --- | --- | --- | --- | --- | --- | --- | --- | --- |
|  | **both** | **male** | **female** | **both** | **male** | **female** | **both** | **male** | **female** | **both** | **male** | **female** | **both** | **male** | **female** | **both** | **male** | **female** | **both** | **male** | **female** |
| 45-46 | 3.3 | 3.8 | 2.8 | 2.8 | 3.1 | 2.4 | 2.7 | 2.5 | 2.9 | 3.2 | 3.4 | 3.0 | 3.4 | 3.3 | 3.5 | 2.0 | 2.3 | 1.7 | 2.9 | 3.0 | 2.7 |
| 47-48 | 3.6 | 3.2 | 4.1 | 3.0 | 3.0 | 3.0 | 2.8 | 3.1 | 2.5 | 3.3 | 3.4 | 3.3 | 3.3 | 3.4 | 3.1 | 2.5 | 3.1 | 2.0 | 3.1 | 3.2 | 2.9 |
| 49-50 | 3.6 | 4.2 | 3.1 | 2.9 | 2.7 | 3.0 | 3.1 | 2.7 | 3.4 | 3.6 | 3.2 | 3.9 | 3.4 | 3.2 | 3.5 | 2.2 | 2.1 | 2.3 | 3.0 | 2.9 | 3.2 |
| 51-52 | 4.2 | 4.8 | 3.7 | 3.5 | 3.5 | 3.5 | 3.0 | 3.2 | 2.9 | 3.7 | 3.7 | 3.6 | 3.5 | 3.7 | 3.3 | 2.9 | 3.4 | 2.5 | 3.4 | 3.6 | 3.1 |
| 53-54 | 3.9 | 3.5 | 4.2 | 3.3 | 3.6 | 3.1 | 3.2 | 3.6 | 2.9 | 3.8 | 4.4 | 3.1 | 4.0 | 3.6 | 4.4 | 2.9 | 3.2 | 2.6 | 3.5 | 3.6 | 3.3 |
| 55-56 | 3.9 | 4.1 | 3.8 | 3.7 | 4.0 | 3.5 | 3.4 | 3.7 | 3.0 | 4.4 | 4.3 | 4.5 | 4.2 | 4.3 | 4.1 | 3.2 | 3.2 | 3.2 | 3.7 | 3.9 | 3.6 |
| 57-58 | 4.1 | 4.4 | 3.9 | 4.6 | 5.5 | 3.6 | 3.7 | 4.0 | 3.2 | 4.3 | 3.9 | 4.6 | 4.1 | 3.8 | 4.5 | 3.4 | 3.3 | 3.4 | 4.0 | 4.1 | 3.8 |
| 59-60 | 4.7 | 4.8 | 4.7 | 4.3 | 4.8 | 3.6 | 3.8 | 3.7 | 3.9 | 4.9 | 5.2 | 4.6 | 5.0 | 4.6 | 5.3 | 3.6 | 3.8 | 3.4 | 4.2 | 4.4 | 4.1 |
| 61-62 | 4.1 | 3.8 | 4.5 | 4.5 | 4.7 | 4.3 | 4.0 | 4.0 | 4.0 | 5.6 | 6.4 | 4.5 | 4.7 | 5.0 | 4.3 | 2.8 | 3.0 | 2.6 | 4.2 | 4.5 | 3.9 |
| 63-64 | 3.9 | 3.6 | 4.2 | 4.9 | 5.1 | 4.6 | 5.5 | 5.5 | 5.4 | 5.0 | 4.7 | 5.3 | 5.6 | 6.0 | 5.0 | 4.3 | 4.5 | 4.2 | 4.8 | 4.9 | 4.7 |
| 65-66 | 6.4 | 5.3 | 7.7 | 5.5 | 6.5 | 4.7 | 5.1 | 5.3 | 4.7 | 6.0 | 6.1 | 5.9 | 5.0 | 5.6 | 4.3 | 5.3 | 5.9 | 4.8 | 5.5 | 5.8 | 5.2 |
| 67-68 | 6.2 | 6.0 | 6.3 | 5.6 | 5.4 | 5.9 | 6.4 | 7.5 | 5.4 | 6.1 | 6.7 | 5.4 | 6.2 | 6.1 | 6.2 | 4.5 | 4.6 | 4.5 | 5.5 | 5.7 | 5.3 |
| 69-70 | 5.6 | 5.5 | 5.8 | 6.4 | 6.4 | 6.5 | 5.6 | 6.2 | 4.7 | 5.3 | 5.6 | 4.9 | 5.2 | 4.4 | 6.0 | 4.8 | 4.6 | 5.0 | 5.3 | 5.2 | 5.3 |
| 71-72 | 6.4 | 6.5 | 6.4 | 6.6 | 8.0 | 5.5 | 6.0 | 5.0 | 7.3 | 5.8 | 6.1 | 5.4 | 6.2 | 6.3 | 6.1 | 6.2 | 5.8 | 6.4 | 6.2 | 6.1 | 6.2 |
| 73-74 | 5.7 | 4.8 | 6.3 | 6.7 | 6.5 | 6.9 | 5.6 | 6.5 | 4.8 | 6.3 | 5.0 | 7.1 | 5.2 | 5.0 | 5.4 | 5.7 | 5.7 | 5.7 | 5.8 | 5.7 | 6.0 |
| 75-76 | 8.0 | 7.3 | 8.5 | 9.5 | 8.1 | 11.4 | 7.4 | 7.6 | 7.1 | 7.5 | 7.5 | 7.5 | 5.8 | 5.3 | 6.2 | 5.7 | 5.2 | 6.2 | 6.9 | 6.4 | 7.4 |
| 77-78 | 6.8 | 7.2 | 6.6 | 8.0 | 10.7 | 6.7 | 6.9 | 5.7 | 8.3 | 6.2 | 5.1 | 7.6 | 7.0 | 8.2 | 6.1 | 6.0 | 5.9 | 6.2 | 6.6 | 6.4 | 6.8 |
| 79-80 | 10.9 | 8.9 | 12.6 | 9.8 | 7.7 | 11.1 | 9.3 | 7.5 | 10.6 | 11.1 | 10.1 | 11.9 | 8.1 | 8.3 | 7.9 | 6.6 | 6.5 | 6.9 | 9.1 | 7.8 | 10.4 |
| 81-82 | 5.0 | 6.5 | 3.5 | 8.6 | 8.2 | 9.1 | 7.7 | 5.9 | 9.1 | 5.2 | 4.4 | 5.6 | 8.0 | 7.7 | 8.2 | 6.8 | 9.2 | 4.8 | 7.0 | 7.4 | 6.7 |
| 83-84 | 13.9 | 12.2 | 16.3 | 9.8 | 16.8 | 5.4 | 7.4 | 5.5 | 9.0 | 7.4 | 2.5 | 10.3 | 8.2 | 7.3 | 8.7 | 6.0 | 4.8 | 6.5 | 8.7 | 8.6 | 8.7 |
| ≥85 | 5.1 | 5.3 | 4.8 | 6.8 | 4.9 | 8.2 | 9.2 | 10.0 | 8.6 | 10.5 | 10.3 | 10.8 | 6.8 | 7.5 | 6.4 | 6.3 | 5.5 | 6.8 | 7.9 | 7.6 | 8.0 |
| total | 3.5 | 3.4 | 3.5 | 3.2 | 3.3 | 3.1 | 3.0 | 3.1 | 2.9 | 3.4 | 3.4 | 3.3 | 3.3 | 3.3 | 3.3 | 3.3 | 3.5 | 3.2 | 3.3 | 3.3 | 3.2 |

Table S4 Hemorrhagic stroke deaths attributable to high sodium intake in different groups from 2010 to 2021

| **Age group** | **2010** | | | **2011** | | | **2012** | | | **2013** | | | **2014** | | | **2015** | | | **2016** | | | **2017** | | | **2018** | | | **2019** | | | **2020** | | | **2021** | | |
| --- | --- | --- | --- | --- | --- | --- | --- | --- | --- | --- | --- | --- | --- | --- | --- | --- | --- | --- | --- | --- | --- | --- | --- | --- | --- | --- | --- | --- | --- | --- | --- | --- | --- | --- | --- | --- |
|  | **B** | **M** | **F** | **B** | **M** | **F** | **B** | **M** | **F** | **B** | **M** | **F** | **B** | **M** | **F** | **B** | **M** | **F** | **B** | **M** | **F** | **B** | **M** | **F** | **B** | **M** | **F** | **B** | **M** | **F** | **B** | **M** | **F** | **B** | **M** | **F** |
| 45-46 | 3 | 1 | 1 | 4 | 3 | 1 | 3 | 1 | 2 | 2 | 1 | 1 | 1 | 1 | 1 | 2 | 1 | 1 | 2 | 1 | 1 | 1 | 1 | 1 | 1 | 1 | 0 | 1 | 1 | 0 | 0 | 0 | 0 | 1 | 1 | 0 |
| 47-48 | 3 | 1 | 1 | 5 | 2 | 2 | 5 | 4 | 1 | 2 | 1 | 1 | 2 | 1 | 2 | 2 | 1 | 0 | 3 | 1 | 1 | 1 | 1 | 1 | 2 | 1 | 1 | 1 | 1 | 0 | 1 | 1 | 0 | 1 | 1 | 1 |
| 49-50 | 3 | 3 | 0 | 2 | 2 | 0 | 3 | 2 | 1 | 3 | 2 | 1 | 3 | 1 | 2 | 2 | 1 | 1 | 2 | 1 | 1 | 2 | 1 | 1 | 2 | 1 | 1 | 2 | 1 | 1 | 1 | 1 | 0 | 2 | 1 | 1 |
| 51-52 | 3 | 3 | 1 | 3 | 2 | 1 | 2 | 1 | 0 | 3 | 2 | 1 | 2 | 1 | 1 | 3 | 2 | 1 | 3 | 2 | 1 | 4 | 2 | 2 | 3 | 2 | 1 | 2 | 1 | 1 | 1 | 1 | 0 | 2 | 1 | 1 |
| 53-54 | 4 | 2 | 2 | 3 | 2 | 1 | 3 | 2 | 1 | 1 | 1 | 1 | 1 | 1 | 0 | 3 | 2 | 0 | 3 | 2 | 1 | 4 | 2 | 2 | 3 | 2 | 1 | 2 | 1 | 1 | 1 | 1 | 0 | 2 | 1 | 1 |
| 55-56 | 5 | 2 | 3 | 5 | 1 | 4 | 4 | 2 | 2 | 3 | 2 | 1 | 3 | 2 | 1 | 2 | 1 | 1 | 1 | 1 | 0 | 2 | 1 | 1 | 3 | 2 | 1 | 4 | 2 | 2 | 2 | 1 | 1 | 3 | 2 | 2 |
| 57-58 | 5 | 4 | 1 | 4 | 4 | 1 | 3 | 2 | 2 | 6 | 4 | 2 | 8 | 6 | 2 | 3 | 2 | 1 | 4 | 2 | 2 | 2 | 1 | 1 | 2 | 1 | 1 | 3 | 2 | 1 | 2 | 2 | 1 | 2 | 2 | 1 |
| 59-60 | 6 | 3 | 3 | 6 | 4 | 1 | 5 | 3 | 2 | 5 | 3 | 2 | 6 | 4 | 1 | 6 | 4 | 2 | 6 | 4 | 2 | 4 | 3 | 1 | 2 | 2 | 1 | 2 | 1 | 0 | 2 | 1 | 1 | 2 | 2 | 1 |
| 61-62 | 4 | 3 | 1 | 4 | 3 | 1 | 4 | 2 | 2 | 4 | 3 | 1 | 6 | 4 | 2 | 6 | 4 | 3 | 5 | 3 | 1 | 4 | 3 | 1 | 3 | 2 | 1 | 3 | 3 | 1 | 1 | 1 | 0 | 2 | 2 | 0 |
| 63-64 | 4 | 2 | 3 | 3 | 2 | 1 | 4 | 2 | 2 | 6 | 3 | 3 | 5 | 4 | 1 | 11 | 9 | 2 | 5 | 3 | 3 | 6 | 3 | 3 | 5 | 4 | 1 | 4 | 3 | 2 | 3 | 2 | 1 | 3 | 3 | 1 |
| 65-66 | 7 | 4 | 3 | 7 | 4 | 3 | 9 | 4 | 5 | 9 | 5 | 5 | 8 | 6 | 2 | 10 | 5 | 5 | 6 | 3 | 3 | 6 | 4 | 2 | 5 | 4 | 1 | 6 | 4 | 2 | 4 | 3 | 2 | 6 | 4 | 2 |
| 67-68 | 9 | 4 | 5 | 6 | 4 | 2 | 8 | 4 | 4 | 10 | 5 | 6 | 8 | 5 | 3 | 9 | 6 | 3 | 11 | 9 | 2 | 11 | 5 | 5 | 7 | 6 | 2 | 6 | 4 | 2 | 5 | 2 | 2 | 6 | 5 | 2 |
| 69-70 | 7 | 2 | 5 | 5 | 2 | 3 | 9 | 5 | 4 | 9 | 4 | 6 | 13 | 8 | 6 | 12 | 8 | 4 | 8 | 5 | 4 | 10 | 4 | 7 | 5 | 3 | 3 | 5 | 3 | 2 | 6 | 3 | 3 | 5 | 3 | 2 |
| 71-72 | 12 | 9 | 3 | 11 | 8 | 3 | 12 | 5 | 7 | 10 | 7 | 4 | 14 | 11 | 3 | 15 | 8 | 8 | 7 | 4 | 4 | 8 | 4 | 4 | 6 | 4 | 2 | 8 | 4 | 3 | 7 | 4 | 4 | 9 | 5 | 4 |
| 73-74 | 14 | 5 | 8 | 8 | 4 | 4 | 16 | 9 | 7 | 11 | 5 | 7 | 15 | 9 | 5 | 12 | 7 | 4 | 11 | 4 | 7 | 8 | 6 | 2 | 8 | 4 | 4 | 10 | 6 | 4 | 8 | 3 | 5 | 8 | 3 | 5 |
| 75-76 | 26 | 11 | 14 | 20 | 9 | 11 | 17 | 9 | 8 | 21 | 13 | 8 | 18 | 9 | 9 | 23 | 10 | 13 | 18 | 14 | 5 | 24 | 6 | 17 | 10 | 5 | 5 | 11 | 5 | 6 | 7 | 4 | 3 | 13 | 7 | 5 |
| 77-78 | 26 | 13 | 13 | 18 | 8 | 10 | 30 | 15 | 14 | 18 | 9 | 9 | 17 | 10 | 7 | 19 | 9 | 10 | 12 | 6 | 6 | 11 | 3 | 7 | 12 | 6 | 6 | 14 | 7 | 7 | 16 | 9 | 8 | 10 | 5 | 4 |
| 79-80 | 42 | 21 | 21 | 23 | 15 | 8 | 35 | 20 | 16 | 40 | 18 | 22 | 17 | 9 | 9 | 21 | 8 | 13 | 29 | 12 | 16 | 25 | 10 | 16 | 18 | 8 | 10 | 17 | 8 | 9 | 15 | 5 | 10 | 17 | 8 | 9 |
| 81-82 | 17 | 12 | 5 | 11 | 5 | 7 | 25 | 15 | 10 | 18 | 13 | 4 | 21 | 10 | 11 | 23 | 8 | 15 | 13 | 5 | 8 | 17 | 11 | 6 | 13 | 6 | 7 | 12 | 5 | 7 | 17 | 10 | 8 | 11 | 6 | 5 |
| 83-84 | 67 | 32 | 35 | 82 | 40 | 42 | 48 | 11 | 36 | 40 | 15 | 25 | 27 | 11 | 16 | 22 | 10 | 12 | 24 | 12 | 13 | 37 | 15 | 22 | 26 | 13 | 13 | 17 | 8 | 9 | 19 | 11 | 8 | 16 | 9 | 7 |
| 85-86 | 16 | 6 | 10 | 18 | 7 | 11 | 13 | 4 | 9 | 24 | 9 | 15 | 21 | 5 | 16 | 26 | 9 | 17 | 24 | 6 | 18 | 11 | 4 | 8 | 20 | 6 | 14 | 17 | 6 | 11 | 24 | 8 | 16 | 14 | 6 | 8 |
| 87-88 | 8 | 2 | 6 | 12 | 4 | 8 | 13 | 4 | 9 | 19 | 6 | 13 | 18 | 3 | 15 | 21 | 6 | 14 | 25 | 6 | 19 | 10 | 4 | 6 | 21 | 5 | 16 | 17 | 5 | 12 | 17 | 5 | 11 | 14 | 5 | 9 |
| 89-90 | 7 | 3 | 5 | 9 | 4 | 5 | 10 | 2 | 8 | 15 | 4 | 10 | 17 | 2 | 14 | 10 | 3 | 7 | 15 | 3 | 12 | 7 | 2 | 5 | 15 | 4 | 11 | 14 | 3 | 10 | 11 | 4 | 7 | 14 | 4 | 10 |
| 91-92 | 4 | 2 | 2 | 5 | 1 | 4 | 6 | 2 | 4 | 8 | 2 | 7 | 7 | 1 | 5 | 7 | 2 | 5 | 15 | 3 | 12 | 6 | 2 | 4 | 10 | 3 | 7 | 10 | 3 | 7 | 11 | 4 | 7 | 10 | 4 | 6 |
| 93-94 | 2 | 1 | 1 | 3 | 1 | 2 | 3 | 0 | 3 | 5 | 1 | 4 | 4 | 1 | 4 | 5 | 1 | 4 | 8 | 1 | 6 | 4 | 1 | 3 | 6 | 1 | 4 | 7 | 1 | 5 | 8 | 2 | 6 | 4 | 1 | 3 |
| 95-96 | 1 | 0 | 1 | 1 | 0 | 1 | 1 | 0 | 1 | 2 | 1 | 2 | 4 | 0 | 4 | 3 | 1 | 2 | 3 | 1 | 3 | 2 | 0 | 2 | 3 | 0 | 2 | 3 | 1 | 2 | 5 | 1 | 4 | 3 | 1 | 2 |

B represents Both gender, M represents Male, F represents Female.

Table S5 Ischemic stroke deaths attributable to high sodium intake in different groups from 2010 to 2021

| **Age group** | **2010** | | | **2011** | | | **2012** | | | **2013** | | | **2014** | | | **2015** | | | **2016** | | | **2017** | | | **2018** | | | **2019** | | | **2020** | | | **2021** | | |
| --- | --- | --- | --- | --- | --- | --- | --- | --- | --- | --- | --- | --- | --- | --- | --- | --- | --- | --- | --- | --- | --- | --- | --- | --- | --- | --- | --- | --- | --- | --- | --- | --- | --- | --- | --- | --- |
|  | **B** | **M** | **F** | **B** | **M** | **F** | **B** | **M** | **F** | **B** | **M** | **F** | **B** | **M** | **F** | **B** | **M** | **F** | **B** | **M** | **F** | **B** | **M** | **F** | **B** | **M** | **F** | **B** | **M** | **F** | **B** | **M** | **F** | **B** | **M** | **F** |
| 45-46 | 0 | 0 | 0 | 0 | 0 | 0 | 0 | 0 | 0 | 0 | 0 | 0 | 0 | 0 | 0 | 0 | 0 | 0 | 0 | 0 | 0 | 0 | 0 | 0 | 0 | 0 | 0 | 0 | 0 | 0 | 0 | 0 | 0 | 0 | 0 | 0 |
| 47-48 | 0 | 0 | 0 | 0 | 0 | 0 | 1 | 1 | 0 | 1 | 0 | 0 | 0 | 0 | 0 | 0 | 0 | 0 | 1 | 0 | 1 | 0 | 0 | 0 | 0 | 0 | 0 | 0 | 0 | 0 | 0 | 0 | 0 | 0 | 0 | 0 |
| 49-50 | 0 | 0 | 0 | 1 | 0 | 0 | 1 | 0 | 0 | 1 | 1 | 0 | 1 | 0 | 1 | 1 | 0 | 0 | 0 | 0 | 0 | 0 | 0 | 0 | 0 | 0 | 0 | 0 | 0 | 0 | 0 | 0 | 0 | 1 | 0 | 0 |
| 51-52 | 1 | 0 | 0 | 0 | 0 | 0 | 0 | 0 | 0 | 1 | 1 | 0 | 1 | 0 | 0 | 1 | 0 | 1 | 1 | 1 | 0 | 1 | 1 | 0 | 1 | 0 | 0 | 0 | 0 | 0 | 0 | 0 | 0 | 1 | 0 | 0 |
| 53-54 | 0 | 0 | 0 | 1 | 1 | 0 | 1 | 0 | 0 | 1 | 1 | 0 | 0 | 0 | 0 | 1 | 0 | 0 | 2 | 1 | 1 | 1 | 1 | 0 | 1 | 1 | 0 | 1 | 1 | 0 | 0 | 0 | 0 | 0 | 0 | 0 |
| 55-56 | 1 | 0 | 1 | 1 | 0 | 1 | 2 | 1 | 0 | 1 | 0 | 1 | 1 | 1 | 0 | 1 | 0 | 0 | 1 | 0 | 0 | 1 | 1 | 0 | 1 | 1 | 0 | 1 | 0 | 0 | 0 | 0 | 0 | 1 | 1 | 0 |
| 57-58 | 1 | 1 | 0 | 1 | 1 | 0 | 1 | 1 | 0 | 1 | 0 | 1 | 3 | 2 | 1 | 1 | 0 | 1 | 1 | 1 | 0 | 2 | 1 | 1 | 1 | 0 | 0 | 1 | 1 | 0 | 1 | 0 | 0 | 1 | 1 | 0 |
| 59-60 | 1 | 0 | 0 | 1 | 1 | 1 | 1 | 1 | 1 | 3 | 2 | 1 | 3 | 2 | 0 | 2 | 1 | 1 | 2 | 2 | 1 | 3 | 2 | 2 | 1 | 1 | 0 | 1 | 1 | 0 | 1 | 0 | 1 | 1 | 1 | 0 |
| 61-62 | 1 | 1 | 0 | 2 | 1 | 1 | 2 | 1 | 1 | 2 | 1 | 1 | 2 | 1 | 1 | 4 | 2 | 2 | 3 | 3 | 1 | 3 | 3 | 1 | 2 | 1 | 0 | 2 | 1 | 1 | 0 | 0 | 0 | 1 | 1 | 0 |
| 63-64 | 1 | 0 | 0 | 1 | 0 | 1 | 2 | 1 | 0 | 2 | 1 | 1 | 3 | 2 | 1 | 6 | 5 | 2 | 3 | 2 | 1 | 4 | 2 | 1 | 3 | 2 | 1 | 3 | 2 | 1 | 1 | 1 | 0 | 2 | 2 | 1 |
| 65-66 | 2 | 1 | 1 | 3 | 1 | 1 | 4 | 1 | 3 | 6 | 3 | 3 | 3 | 3 | 1 | 5 | 3 | 2 | 4 | 2 | 1 | 6 | 4 | 2 | 4 | 2 | 1 | 2 | 1 | 1 | 3 | 3 | 1 | 2 | 2 | 1 |
| 67-68 | 2 | 1 | 1 | 2 | 1 | 1 | 5 | 2 | 3 | 8 | 3 | 5 | 4 | 3 | 1 | 8 | 6 | 2 | 8 | 6 | 2 | 9 | 5 | 4 | 3 | 2 | 1 | 4 | 3 | 1 | 3 | 2 | 1 | 4 | 2 | 2 |
| 69-70 | 2 | 1 | 1 | 3 | 1 | 2 | 4 | 2 | 2 | 7 | 2 | 4 | 9 | 4 | 6 | 9 | 5 | 4 | 6 | 4 | 2 | 8 | 3 | 4 | 5 | 3 | 2 | 4 | 3 | 2 | 3 | 2 | 1 | 4 | 3 | 2 |
| 71-72 | 3 | 2 | 1 | 4 | 4 | 1 | 8 | 6 | 3 | 6 | 4 | 2 | 10 | 7 | 3 | 12 | 4 | 8 | 7 | 3 | 3 | 8 | 4 | 4 | 7 | 4 | 3 | 7 | 5 | 3 | 6 | 3 | 2 | 7 | 4 | 3 |
| 73-74 | 4 | 2 | 3 | 4 | 2 | 2 | 9 | 5 | 4 | 9 | 4 | 5 | 11 | 6 | 5 | 12 | 7 | 5 | 11 | 5 | 6 | 9 | 6 | 3 | 7 | 4 | 3 | 6 | 3 | 3 | 7 | 3 | 4 | 7 | 4 | 3 |
| 75-76 | 14 | 4 | 11 | 15 | 7 | 8 | 13 | 5 | 8 | 17 | 10 | 6 | 19 | 8 | 11 | 25 | 14 | 11 | 20 | 14 | 7 | 30 | 8 | 21 | 9 | 4 | 5 | 12 | 6 | 5 | 7 | 4 | 2 | 9 | 5 | 5 |
| 77-78 | 13 | 5 | 8 | 11 | 5 | 6 | 27 | 12 | 15 | 20 | 11 | 9 | 20 | 9 | 10 | 24 | 11 | 13 | 17 | 7 | 10 | 16 | 5 | 12 | 13 | 8 | 5 | 12 | 6 | 6 | 13 | 8 | 6 | 10 | 5 | 5 |
| 79-80 | 18 | 7 | 11 | 17 | 11 | 5 | 30 | 16 | 14 | 39 | 16 | 22 | 20 | 10 | 9 | 30 | 12 | 19 | 37 | 16 | 20 | 44 | 17 | 27 | 19 | 9 | 10 | 19 | 8 | 11 | 15 | 7 | 8 | 16 | 7 | 9 |
| 81-82 | 5 | 3 | 2 | 9 | 3 | 6 | 24 | 15 | 9 | 18 | 14 | 4 | 29 | 13 | 16 | 29 | 10 | 19 | 25 | 9 | 16 | 26 | 13 | 12 | 17 | 6 | 10 | 14 | 6 | 8 | 26 | 15 | 10 | 13 | 6 | 7 |
| 83-84 | 34 | 16 | 18 | 54 | 25 | 29 | 40 | 11 | 29 | 39 | 13 | 26 | 27 | 17 | 11 | 34 | 14 | 20 | 35 | 18 | 18 | 72 | 30 | 42 | 28 | 13 | 14 | 26 | 13 | 13 | 30 | 16 | 13 | 24 | 12 | 12 |
| 85-86 | 7 | 4 | 3 | 14 | 5 | 9 | 14 | 4 | 9 | 23 | 8 | 14 | 33 | 8 | 25 | 36 | 13 | 24 | 45 | 12 | 34 | 24 | 8 | 16 | 26 | 8 | 17 | 33 | 11 | 22 | 33 | 13 | 20 | 22 | 9 | 14 |
| 87-88 | 5 | 1 | 3 | 10 | 4 | 6 | 9 | 3 | 7 | 20 | 8 | 13 | 24 | 5 | 20 | 32 | 10 | 22 | 42 | 11 | 31 | 21 | 8 | 13 | 33 | 11 | 22 | 25 | 9 | 16 | 25 | 10 | 15 | 24 | 9 | 15 |
| 89-90 | 3 | 1 | 2 | 5 | 2 | 3 | 7 | 2 | 5 | 16 | 4 | 12 | 16 | 4 | 13 | 22 | 6 | 16 | 22 | 6 | 16 | 16 | 5 | 11 | 20 | 4 | 15 | 24 | 8 | 16 | 21 | 8 | 13 | 21 | 7 | 14 |
| 91-92 | 2 | 1 | 2 | 4 | 1 | 4 | 6 | 1 | 5 | 9 | 3 | 6 | 11 | 3 | 8 | 16 | 5 | 11 | 21 | 4 | 17 | 11 | 3 | 9 | 16 | 3 | 13 | 17 | 3 | 15 | 19 | 5 | 13 | 17 | 4 | 13 |
| 93-94 | 1 | 0 | 1 | 3 | 1 | 2 | 3 | 1 | 2 | 5 | 1 | 4 | 7 | 2 | 5 | 7 | 2 | 5 | 12 | 2 | 10 | 7 | 2 | 5 | 8 | 1 | 7 | 9 | 2 | 8 | 9 | 2 | 7 | 12 | 3 | 9 |
| 95-96 | 1 | 0 | 1 | 1 | 0 | 1 | 1 | 0 | 1 | 3 | 1 | 2 | 4 | 1 | 3 | 2 | 0 | 2 | 5 | 1 | 4 | 3 | 1 | 2 | 5 | 1 | 4 | 7 | 2 | 5 | 6 | 1 | 5 | 7 | 2 | 5 |

B represents Both gender, M represents Male, F represents Female.
